# Reconceiving Domestic Burning Controls: Air Quality Alerts, Behavioural Responsive Regulation, and Designing for Compliance

**DOI:** 10.1007/s00267-024-02014-z

**Published:** 2024-08-09

**Authors:** James Heydon, Rohit Chakraborty, Vibhuti Patel, Chantelle Wood, Matthew Wood, Caitlin Bunce

**Affiliations:** 1 University of Nottingham, Nottingham, UK; 2 University of Sheffield, Sheffield, UK

**Keywords:** Air quality, Air pollution, Domestic combustion, Behaviour change, Behavioural responsive regulation, Compliance

## Abstract

Domestic combustion emissions pose a growing risk to public health, especially in the UK. Existing responses are polarised, with government advocating use of lower emission fuels and stoves while clean air campaigners call for blanket bans on burning. However, each approach is limited in its ability to control these emissions. An alternative can be found in the U.S.A., where ‘burn alert’ systems require stove and fireplace users to avoid lighting during periods of actual or projected poor air quality. Given the effectiveness of these regimes, the current study designs and evaluates the effectiveness and acceptability of a burn alert system in the UK for the first time, drawing on the theoretical perspective of behavioural responsive regulation. Fifty participants were recruited to use the system over 2 weeks in winter. The findings illustrate that a voluntary burn alert system can dissuade burning among users. Of those in receipt of an alert, 74% reduced burning frequency or burned for a shorter duration. In total, the alert system prevented at least 178 hours of burning for this group. Qualitative findings show that the consistency of the behavioural response is influenced by technical, structural, and environmental factors, providing key insight into how UK-based burn alert systems could be modified to increase the consistency of compliance in future. The overall conclusion is that burn alerts could be introduced in the UK and beyond, as a means of reducing domestic combustion emissions and their associated public health risks.

## Introduction

Air pollution ranks alongside climate change as the biggest environmental threat to human health (World Health Organization WHO 2021), becoming a political priority for institutions at the international (UN Environment Programme 2023), regional (European Environment Agency 2023), and national levels (US Department of State 2023). One of the most harmful components of air pollution is fine particulate matter (PM2.5). Small enough to bypass bodily defences and infiltrate every organ (Schraufnagel et al. 2019), there is no known ‘safe’ limit of exposure (Brauer et al. 2022). PM2.5 increases the risk of respiratory infections across age groups (Yang et al. 2020) and is associated with the onset of asthma in children, and lung cancer, dementia, and Parkinson’s disease in adults (Garcia et al. 2023; Li et al. 2022). Globally, the two decades between 1990 and 2019 saw the number of deaths attributable to ambient PM2.5 increase by 102.3%, contributing to a mortality rate of over 4 million people per year (Sang et al. 2022: 1).

PM2.5 can be produced through non-anthropogenic means, such as windblown mineral dust from arid regions and forest fires (see McDuffie et al. 2021), but one of the dominant sources is the domestic burning of solid fuel for heating. This is a problem in population centres across Europe (Kortekand et al. 2022; Maione et al. 2021), North America (Rokoff et al. 2017), Australasia and beyond (Kushel et al., 2022; Oliver et al. 2020). The UK is no exception. As reported by the Department for Environment, Food and Rural Affairs (Defra), domestic combustion produces 21% of all PM2.5 emissions nationally (Defra 2023a). This is the primary source of these emissions, with road transport second at 13% (Defra 2023a). Yet, despite increasing political and scientific awareness, the problem of domestic burning is getting worse. In the decade between 2011 and 2021, PM2.5 emissions from domestic combustion increased by 124% (Defra 2023a), compelling the UK Government to include a commitment to their reduction in its long-term Environmental Improvement Plan (HM Government 2023).

The UK’s current approach to reducing PM2.5 from domestic combustion centres on persuading stove and fireplace users to ‘burn better’. The main instrument for achieving this is the ‘smoke control area’ (SCA); geographical regions where residents are prohibited from emitting visible smoke at risk of a warning and subsequent civil penalty (Defra 2022). Advocating a move away from high smoke fuels, like coal, and onto dry wood and newer, lower emission stoves (Defra 2023b), this strategy has several limitations. Stove upgrades are costly and their effectiveness uncertain; improving air quality in some cases (Noonan et al. 2011) but not others (Lopez-Aparicio and Grythe 2020; Robinson 2016). Even under ideal conditions, the latest Ecodesign stove standard permits appliances to emit 375 g of PM2.5 for every gigajoule (GJ) of energy produced—some 750 times that of a heavy goods vehicle (European Environmental Bureau 2021: 7). These stoves also emit twice the amount of ultrafine particulate matter as older designs (Kuye and Kumar 2023), which is smaller and more toxic than PM2.5 (Schraufnagel 2020). Ultimately, in its effort to reduce smoke, the UK’s approach encourages behaviour that still produce abundant amounts of PM2.5 (Heydon 2023; Horton and Harvey 2023).

Regulatory systems that exert more direct control over PM2.5 from domestic combustion originate in the U.S.A. Known as ‘burn alert’ systems, these go further than encouraging people to ‘burn better’ by issuing temporary bans on burning activity during actual or projected high particulate pollution episodes (see Appendix Table  4). Contravening this instruction triggers an escalation of sanctions leading up to a fine; an approach widely associated with emissions reduction and benefits for health. For example, compliance with the Bay Area Air Quality Management District (BAAQMD) system caused particulate pollution to fall between 11 and 15 percent in its first year, while the number of elderly people admitted to hospital with heart problems decreased by between 7 and 11 percent (Yap and Garcia 2015: 772). Of the ten ‘no burn days’ called by the South Coast Air Quality Management District in 22/23, alerts prevented a breach of air quality thresholds on seven occasions (Rees 2023). Similar success was evidenced with regimes in Washington, which saw levels of PM pollution return to federally acceptable limits within 3 years (Department of Ecology 2014). Despite these results, similar systems have yet to be introduced outside the U.S.A.

The current study examines whether burn alerts could work to dissuade domestic burning in a UK context, examining their ability to change burning behaviour. Drawing on the theoretical perspective of behavioural responsive regulation (BRR) devised by Barak-Corren and Kariv-Teitelbaum (2021), this study designs and evaluates the effectiveness and acceptability of a burn alert system in the UK for the first time. The purpose and significance of this research is three-fold. Firstly, by walking a path between the permissive ‘burn better’ approach of government (Defra 2023b) and the more adversarial ‘bans’ advocated by clean air campaigners (see Mums for Lungs 2023), the study aims to explore the opportunities for—and obstacles to—successful introduction of a voluntary burn alert system enabling the co-existence of the two positions. In doing so, it contributes evidence of a possible alternative within an otherwise polarised discussion on the solutions available to a pressing environmental and public health issue.

Secondly, the current study has clear potential to influence policy. If the burn alert system is effective and acceptable, this intervention could drive an update of the UK’s domestic combustion regulations to directly intervene in PM2.5 emissions. Introduced in the 1950s to address London’s smog episodes, SCAs were originally aimed at dissuading people from burning house coal which produces a large amount of visible smoke (see Corton 2015). As such, SCAs were not designed to prevent all burning, only that which produces smoke. However, advances in sensor technology—made since the 1950s—mean that PM2.5 (which can be emitted without visible smoke) is now recognised as a key health issue. SCAs are therefore increasingly outdated in a context where national and international commitments to reducing PM2.5—and other pollutants—are advancing at pace (see Maltby 2022). Yet, despite their difference in focus, the enforcement hierarchies of SCAs broadly mirror those of the burn alert systems (see Appendix Table 4). This presents a unique opportunity to pivot the UK’s regulatory apparatus towards the more contemporary problem of PM2.5. The present research therefore provides a timely answer to the ‘principal dilemma for policymakers’ as it relates to domestic combustion; not ‘*whether* to regulate, but *what* and *how* to regulate’ (Barak-Corren and Kariv-Teitelbaum 2021: 168).

The final purpose of this article is to demonstrate the application of theory. BRR integrates two influential theories of regulation: responsive regulation (RR) and behavioural public policy (BPP). Drawing insight from the latter to improve compliance with rules established by the former, the resulting framework makes the theoretical prescription to ‘design for compliance’ (Barak-Corren and Kariv-Teitelbaum 2021: 172). Emphasising the need to create regulatory settings that ‘facilitate honest behaviour and compliance with obligations, prior to any further attempt to apply either soft or coercive measures’ (Barak-Corren and Kariv-Teitelbaum 2021: 173), this approach orientates research to engagement with interventions outside sanction hierarchies. It is this aspect of BRR that is advanced here. Given that ‘burn alerts’—enforced or otherwise—are yet to exist in the UK, this study represents the ‘formative evaluation’ of one such system; a pilot of a novel and innovative intervention that can lead into larger randomised control trials (Wight et al. 2016: 524). This avoids the costly introduction or assessment of unpromising interventions, forming a critical step in evidence-based design (Beets et al. 2021). By trialling user engagement with a voluntary burn alert tool, this article breaks new ground by demonstrating how BRR’s theoretical prescription to ‘design for compliance’ can be operationalised in practice.

The article proceeds in four sections. Air Quality Alert Systems: Designing for Compliance explores BRR and its instruction to ‘design for compliance’, using this to draw lessons from the behavioural research on air quality information and environmental warning systems. Intervention Design details the system design and research methodology. The subsequent Findings and Analysis section presents data on changes in behaviour resulting from the intervention, in addition to data on user experiences with and acceptability of the burn alert system itself. The final section, Discussion, draws out the implications of these results for future burn alert systems, exploring how their integration with the UK’s existing regulatory regime could provide a pathway for more direct control of PM2.5 emissions into the future.

## Air Quality Alert Systems: Designing for Compliance

The purpose of BRR is to integrate RR and BPP to improve the effectiveness, efficiency, and legitimacy of regulations (Barak-Corren and Kariv-Teitelbaum 2021: 176–178). RR emphasises the need for discretion and dynamism among regulators, advocating for their iterative movement between coercion and persuasion depending on regulatee behaviour (Ayres and Braithwaite 1992). The widely deployed pyramid of enforcement strategies captures this key premise (see Ivec et al. 2015), recommending that interventions escalate in severity until compliance is achieved. Moving from dialogue-based responses at the base of the pyramid (e.g. persuasion and warning letters) to more coercive and deterrence-based sanctions at the apex (e.g. civil and criminal penalties), this strategy of escalation in the event of non-compliance is intended to force most regulatory action towards the lower levels of the pyramid, avoiding the costly approach of uniformly applying stringent sanctions (Braithwaite 2011).

Contrasting with the explicit role of penalties in RR, BPP structures decision-making environments to encourage behaviour change without the threat of sanctions. Drawing on behavioural science in the design and application of rules and their administration, BPP encompasses a range of theories and frameworks. One of the most prominent is the COM-B Model (Michie et al. 2011), which argues that for a behaviour to occur, people must have the capability, opportunity, and motivation to engage in that behaviour. As such, interventions that seek to change behaviour require modification of at least one of these determinants, drawing on appropriate behaviour change techniques to do so (e.g., see Michie et al. 2013).

In modifying RR’s hierarchic pyramid with BPP’s non-hierarchic toolbox, BRR draws these two perspectives together. This is done in two stages. First, behavioural tools are used to design a new layer at the base of the pyramid with the express purpose of facilitating compliance. This speaks to the regulatory environment itself, requiring features that help regulatees adhere to rules without the threat of sanction (e.g. simplifying tax forms and minimising the effort required to complete them) (Barak-Corren and Kariv-Teitelbaum 2021). The second stage of integration asks that behavioural insights from BPP inform every subsequent layer of the pyramid. The purpose of this is to make regulatory interventions more effective at encouraging compliance once triggered (e.g. improving the educational potential of advisory letters and making warnings more persuasive). Given that burn alert systems have yet to exist in the UK, the first theoretical prescription is of primary concern here—to ‘design for compliance’ (Barak-Corren and Kariv-Teitelbaum 2021: 172).

Designing for compliance involves the creation of regulatory settings that facilitate obedience with rules ‘prior to any act on the part of regulatees’ (Barak-Corren and Kariv-Teitelbaum 2021: 171–172). It is primarily concerned with encouraging people to ‘do what the regulations stipulate that they should do’ before sanctions are applied (Six et al. 2023: 5). There exist multiple motivations for this observance of rules, irrespective of their legality, ranging from rational calculation or belief in regulatory ‘wisdom’, through to social conformity or moral identification with the law (Feldman 2011; Murphy et al. 2009; Tyler 2006). As these are not mutually exclusive (Winter and May 2001), behavioural interventions should seek to ‘target as many motivations as possible’ (Feldman 2011: 343). This is where behavioural research has influenced the regulatory literature, advocating for an attentiveness to intervention presentation, and ‘to the values, norms and assumptions that the presentation communicates’ (Barak-Corren 2022: 1094).

Emphasising intervention characteristics and user experiences of them, the focus on presentation is of central concern to research aimed at evaluating the two foundational components of ‘burn alerts’: air quality information systems and their environmental warning counterparts. As voluntary endeavours are unable to evoke the more ‘instrumental’ motivations associated with sanction threats (Tyler 2006), these systems have prompted a rich literature aimed at understanding how rule observance can be facilitated without coercion. Taking the work on behavioural responses to air quality information systems as the point of departure, its lessons are diverse. The research explores the role of different sensing technologies, communication mediums and data presentation formats on a variety of actors, all of which are aimed at inducing behaviours ranging from pollution reduction and exposure avoidance through to increasing engagement with environmental issues (Workman et al. 2021; D’Antoni et al. 2019; Mehiriz and Gosselin 2019; Oltra and Sala 2015). The obstacles to behaviour change are similarly varied, speaking to the perceived credibility of data sources (Riley et al. 2021), presentation and content of messages (Heydon and Chakraborty 2022), appropriateness of communication mediums (Workman et al. 2021), and receiver characteristics, all of which are implicated in intervention success or failure.

The equivalent literature on warning systems for heat, drought, and flooding is equally instructive. Here, obstacles to behaviour change originate in poor information visualisation, inappropriate messaging language, poor location search functionality and unintuitive interfaces (Khamaj and Kang 2018). Uncertainty induced by multiple alert systems also affects user engagement, as does data lacking local-level granularity and warnings that do not communicate enough about health effects (Roberts et al. 2022). One of the most prominent challenges to compliance is ‘warning fatigue’ (Potter et al. 2021: 307). Arising when users are exposed to a large volume of irrelevant or repeated alerts over long periods (McLean et al. 2018; Weinberger et al. 2018; Rayo and Moffatt-Bruce 2015), the ensuing ‘fatigue’ causes users to become overwhelmed and unresponsive to instructions. Using Riley et al.’s (2021) COM-B-informed model, the challenges to and recommendations for designing both types of system ‘for compliance’ have been combined in Table 1. As explained in the next section, this was used to design the burn alert system under evaluation here.

**Table 1:** Designing for compliance—lessons from the air quality information and environmental warning literatures

Communication dimension	Challenges	Recommendations
Message source	Public disengage with information deemed partisan, compromised, commercial and emphasising individual responsibility; Multiple alert systems induce uncertainty (Roberts et al. 2022)	Establish a trusted and credible source; Demonstrate collective responsibility; Ensure source is perceived as legitimate (Wood et al. 2023); Data should be accurate, timely, meaningful and geographically specific (Tarchiani et al. 2020)
Message content	Singular behavioural responses minimise prospects for change; Low self-efficacy inhibits belief in options for behavioural change; Halo effect; Broad, general, numeric information and indices unlikely to engage people (also Heydon and Chakraborty 2022); High message frequency can induce ‘warning fatigue’ (Potter et al. 2021; Weinerger et al. 2018; Rayo and Moffatt-Bruce 2015)	Messages should be human-centred, use positive framing and avoid alarmism; Use personification, storytelling, and metaphor; Connect with peoples’ emotions; Simple but not simplistic information; Relatable, understandable, and local information; Communicate co-benefits of action; Encourage supportive actions and communicate a range of actions; Connect people to collective action; Explain thresholds/indices and avoid over-quantification; Include clear and specific guidance/instructions (Roberts et al. 2022); Ensure lead-time for behaviour change (Roberts et al. 2022); Communicate impacts associated with environmental conditions (Potter et al. 2021; (Kaltenberger et al. 2020)
Communication channel	Mass channels unlikely to deliver local, positive, personal and targeted messages (also Mehiriz and Gosselin 2019); Poor visualisation of information, language, location search functionality and menu systems (Khamaj and Kang 2018)	Appropriate to deliver the message; Communicate beyond high pollution episodes; Targeted smartphone alerts where appropriate (Workman et al. 2021; Mehiriz and Gosselin 2019); Interface should be intuitive, aesthetic, and concise (Khamaj and Kang 2018)
Information recipient	Impersonal communication can discourage engagement; Alerts assuming prior understanding (Roberts et al. 2022)	Activate social norms and identities; Tailor information to the receiver (personal and specific) (also Tarchiani et al. 2020) Supplement with wider awareness-raising initiatives (Heydon and Chakraborty 2022; Tarchiani et al. 2020)

Adapted from Riley et al. (2021: 2040–2043)

## Intervention Design

**Fig. 1:**
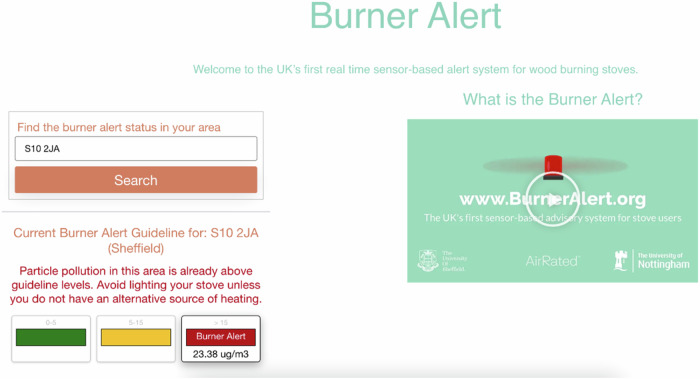
‘Burner Alert’ webpage showing red alert notification

The UK’s first ‘Burner Alert’ (BA) system was co-designed with air quality scientists at Air Rated. The system could be accessed via desktop computers, tablets, and smartphones, and was compatible with Android and iOS devices. It did not have an app, but when accessed via a smartphone gave the option of installing a home screen shortcut to open the website. Following evidence that ‘public perceptions of pollution are grounded in local places, local hotspots and local sources’ (Riley et al. 2021: 2042), the website invited users to input their postcode and receive information on air pollution at the given location (see Fig. 1).

An animation was created to accompany the system (Sheffield:Air 2022). This provided information on PM2.5, its health effects, and guidance on how to interpret the alerts. Following research illustrating the need to be explicit about health impacts (D’Antoni et al. 2019), children, the elderly and those with pre-existing conditions were highlighted as particularly vulnerable. This was also intended to engage emotions (Riley et al. 2021). In explaining each alert threshold, emphasis was placed on communicating meaning beyond exposure (Haddad and de Nazelle 2018), avoiding over-quantification (Heydon and Chakraborty 2022), and providing specific guidance on the actions required (Roberts et al. 2022). In each facet of this system—animation, information, and alerts—neutral language was deployed to avoid alarmism and negative framing (Riley et al. 2021). The amount of information displayed at any one time was minimised, brevity of instructions prioritised, and the postcode search function centred, heeding evidence that interfaces should be intuitive, aesthetic, and concise (Khamaj and Kang 2018).

The World Health Organization’s 2022 guideline values for PM2.5 pollution over a 24-h period were adopted as the basis for the three alert thresholds (see Table 2). This was chosen to avoid the controversy around the UK Government’s higher thresholds (Harvey 2022), acknowledge that health effects result from even low levels of exposure (Brauer et al. 2022), and provide a trusted source to encourage message acceptability (Wood et al. 2023; Walker et al. 1998). Actionable information accompanied each alert, to fulfil its function of dissuading stove users from lighting during periods of high or moderate air pollution. Colour coding was used to indicate risk levels, though this was accompanied by the quantity of PM/ug3 and a brief explanation to ensure the guidance was ‘simple but not simplistic’ (Riley et al. 2021: 2040).

**Table 2:** Alert thresholds and corresponding recommendation messages

Threshold	Alert text and colour	Alert message
0–5 ug/m^3^	No alert (Green)	Particle pollution in this area is well below guideline levels. Air quality is not currently unhealthy, although stove use may increase the levels in your area.
5–15 ug/m^3^	Advisory (Amber)	Particle pollution in this area is approaching guideline levels. Please consider not lighting your stove, particularly if you have an alternative source of heating.
>15 ug/m^3^	Burner alert (Red)	Particle pollution in this area is already above guideline levels. Avoid lighting your stove unless you do not have an alternative source of heating.

A spatiotemporal convolutional Long Short-Term Memory (LSTM) model was used to predict air pollutant concentrations. This leveraged hourly PM2.5 datasets collected from the national Automated Urban and Rural Network (AURN) operated by Defra. LSTM models are effective in predicting air pollutant concentrations, including PM2.5, by considering historical air pollutant and meteorological data. These models forecast pollutant levels at locations where measurements are not directly available and are widely used in comprehensive air monitoring where sensor networks are unevenly distributed (Wang et al. 2021). The AURN dataset for PM2.5 was created in 2009 (see Defra 2024), providing the model with over a decade of historical data for the burn alert readings.

## Methods

For 6 months between the 9th of October 2022 and 9th March 2023, 50 stove or fireplace users were recruited to test the BA system. This period was chosen because it coincides with the UK’s domestic combustion season (Kantar 2020). Participants were recruited using social media and university staff email lists, with those interested asked to complete a pre-use survey before accessing the website. This survey gathered information on their demographics, burning behaviour, perspectives on air quality and knowledge of domestic combustion regulations.

After submitting their answers, participants were emailed the web address to the BA, asked to watch the instructional animation, and subsequently access the system whenever they considered lighting their appliance. A post-use survey was then administered after 2 weeks of use, to gather data on how the system was used and its effects on burning behaviour, if any. This second survey had a greater proportion of open-text questions than the first, as it was aimed at understanding why the system had been used in particular ways and identifying opportunities for design improvements. To do this, the post-use survey questions measured aspects of usability and acceptability; both of which influence engagement with interventions (Lyon et al. 2021; Sekhon et al. 2017).

Jisc Surveys was used to administer the surveys, while SPSS and Microsoft Excel were used to analyse the quantitative data. Braun and Clarke’s (2006) approach to thematic analysis was adopted alongside Fereday and Muir-Cochrane’s (2006: 80) ‘hybrid’ coding position, allowing for the influence of both inductive and deductive reasoning in the analysis of open text responses (see Braun and Clarke 2021). Priority was given to the analysis of qualitative responses in the context of the closed questions to which they related, being used to ‘enhance, confirm [and] refine’ the story told through the quantitative data (Rouder et al. 2021: 3). Participation was contingent on the provision of free, prior, and informed consent, with all participants entered into a £20 prize draw on completion of both surveys. Ethical approval was granted by the Ethics Sub-Committee at the University of Nottingham, reference no. 2021-083-STAFF.

### Participant Sample

Participation was restricted to those using stoves and fireplaces as a secondary heat source. This was because the study occurred in winter 2022/23, when increased fuel prices drew many UK residents into ‘fuel poverty’ (Hinson and Bolton 2023). This decision was taken to minimise the risk of alerts encouraging those without alternatives (around 5% of stove users (Kantar 2020: 11) to avoid heating their home. Sample characteristics can be viewed in Table 3.

**Table 3:** Sample characteristics (*n* = 50)

Characteristic	Domestic burners
Age	*M* = 42.42 (SD = 11.43)
Gender	48% female; 46% male; 4% gender variant/non-conforming; 2% prefer not to say
Ethnicity	98% white; 2% mixed/multiple ethnic groups
Children in the home	70% without children; 30% with children
People in household with respiratory Condition	70% none; 12% partner; 8% respondent; 6% respondent and child; 2% child; 2% partner and child
Area type^a^	42% city; 46% rural; 12% urban
Geographical spread	Across 18 counties; 54% from Yorkshire and Derbyshire
Appliance type	94% stove; 6% open fireplace
In a smoke control area	36% no; 32% yes; 32% I don’t know

^a^Categories defined by Defra (2016: 3)

## Findings and Analysis

### Compliance

**Fig. 2:**
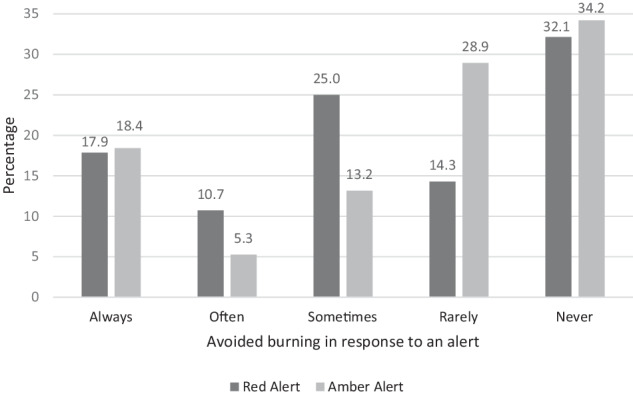
Avoidance responses by Alert Type. *Excludes ‘never received’ values (see Appendix Table 6)

Over a 2-week period, 92% (*n* = 46) of users received an amber and/or red alert. Of these, 74% (*n* = 34) responded by reducing their burning frequency at least once (*n* = 33) or burning for a shorter duration (*n* = 1). Nineteen of the 33 users that reduced burning frequency quantified the number of times an alert prevented lighting: a total of 65 occasions. Accounting for the duration users reported lighting for, the alert system prevented 178 hours of burning for this group. Additionally, green alerts did not have the counterintuitive effect of encouraging users to light; 92% (*n* = 46) were not persuaded to burn when they otherwise would not have done. While most users chose not to burn at least once on receipt of an amber or red alert, this was not a uniform reaction. Inconsistency was a defining feature of alert response, with most users following some alerts and not others at different times (see Fig. 2; Appendix Table 5).

Two dominant reasons were given for consistent compliance with recommendations to avoid burning. Those following red alert advice referred to the adverse personal, social and health consequences of lighting. Users ‘feeling bad’ (User 4), not liking the ‘thought of my children’s lungs being polluted and damaged’ (User 37) and wanting to be ‘a good citizen’ (User 22), all discouraged participants from burning on receipt of an alert. These justifications were mirrored by those avoiding lighting on receipt of an amber alert, but these users also cited mild weather conditions as giving the impetus to act on alert recommendations. For instance, User 1 noted that ‘it wasn’t so cold that we felt it necessary to light…and the amber alert gave us extra motivation not to’, and User 2 described not needing ‘a fire or heating as the weather has been mild’.

As responses become more inconsistent, other justifications for not following recommendations were offered. Here, similar personal, social and health justifications were still present, but factors associated with fuel costs and the less rigid nature of amber alert recommendations also exerted influence. Users described alerts as inducing feelings of conflict when viewed in the context of winter temperatures and energy prices. As User 43 noted, ‘I felt guilty, but when I did light it, I was trying to save on energy bills’. Similarly:

*It did make me hesitate/delay lighting the fire on occasion, but it is our only affordable and the most effective way of heating our home currently. If we had a cheaper alternative, I do think it would have made me avoid lighting the fire more*.

(User 39)

*It was so cold during this period (snow/ice on the ground) that we needed the additional heating. The burner alert did make me think about it though, and if it hadn’t been so cold I would have tried to use it less*.

(User 48)

Such explanations draw attention to the role of structural and environmental factors in either encouraging or discouraging users to follow alert guidance. The other dominant justification for inconsistently avoiding burning was given in specific relation to amber alerts. As these were less forthright in their recommendations to avoid burning, users would consider where their air quality reading sat in relation to the red alert threshold and use that to decide whether to light. As User 22 noted, ‘I made a judgement. If I was at the low end I might light, at the higher end I decided not to’. Similarly:

*If it was close to the tipping point, I’d avoid lighting a fire because I didn’t want to contribute to the fire making air quality go to dangerous levels. If it was low in the amber alert, I’d consider lighting it*.

(User 35)

Others also spoke of the ‘vanilla’ nature of the amber alert (User 16), where its request to ‘consider not lighting’ was taken to ‘suggest that you can light…but use the fire sparingly’ (User 49). This was a common justification for those that ‘rarely’ avoided burning during an amber alert; its language is permissive relative to its red counterpart.

Finally, the most common justification for ‘never’ avoiding burning referred to two aspects of the system itself; alert frequency and modelling granularity. Alerts that did not deviate from red or amber caused some users to doubt that their actions could improve air quality anyway, reflecting low self-efficacy (Riley et al. 2021). As User 26 noted, ‘I live on a main road which is highly polluted due to traffic. Not using my burner would make a negligible difference’. Similarly:

*I checked the burner alert website multiple times over the two weeks, and it was always red, whatever time of day. I came to the conclusion that living in an inner-city area, very close to an incinerator, it was unlikely we’d ever be green or amber*.

(User 19)

For other users, the lack of alert variance raised questions about the monitoring sensitivity underpinning air quality readings. This caused some to cross-reference the figures with other localised networks or their own sense perception:

*[E]very single time I checked bar once, the alert was set on amber. I was not informed where the sampling device was located relative to my post code so these conditions didn’t motivate me to believe I would really make a difference when the air near my house felt really fresh*.

(User 33; also 5; 7)

Taken together, the unchanging nature of some alerts induced a specific form of alert fatigue amongst recipients. Here, the effect was not to be overwhelmed by warning recommendations (see Roberts et al. 2022), but to question the validity of the data on which they were based. This inhibited compliance during use, but also posed a challenge to compliance over time by undermining confidence in the system. This question of data accuracy was a primary explanation for the 42% of participants saying they would ‘maybe’ continue using the system after the study had ended (40% ‘yes’, 16% ‘no’). The other related to memory; users described the process of manually checking the BA as requiring effort, stating that continued use would be more likely if alerts were communicated using app-based push notifications.

### Intervention usability and acceptability

**Fig. 3:**
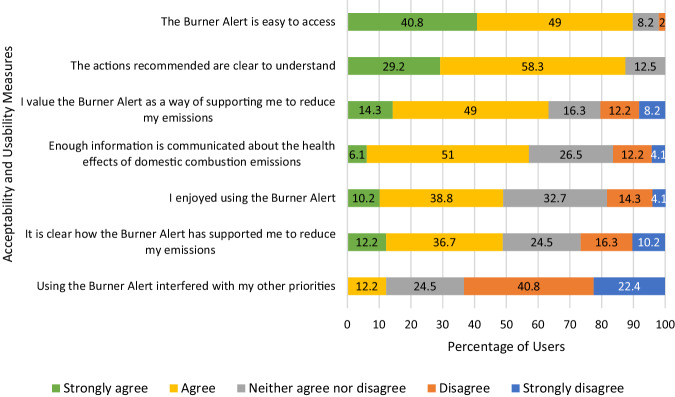
Intervention acceptability and usability

Across most measures (see Fig. 3), responses demonstrated a high degree of intervention acceptability and usability, with participants indicating where modifications to the system itself could improve measures of both.

With the majority accessing the system via smartphone (68%) or desktop computer/laptop (26%), most users considered the BA ‘easy to access’ (90% ‘strongly agree’ and ‘agree’). Most participants (92%) also reported the lack of effort this entailed, with the majority considering the system to take ‘no effort’ (28%) or ‘hardly any effort’ (64%) to use. Similarly, for over two thirds of users (63%), the BA did not interfere with other priorities. Of the 12% reporting interference, most of the priorities mentioned related to cost and warmth. Over two thirds of users (14% ‘strongly agree’ and 49% ‘agree’) also valued the BA as a way of supporting them to reduce emissions, and for almost half of users (12% ‘strongly agree’ and 37% ‘agree’), it was clear how the BA had supported them in this goal.

Almost half (10% ‘strongly agree’ and 39% ‘agree’) of users considered the BA enjoyable to use, largely because it provided ‘interesting’ and ‘useful’ information about air quality in their local area (Users 6; 11, 12; 14; 16; 17; 23; 27; 30; 34; 38; 39; 49). This perspective was shared by many of the 33% answering ‘neither agree nor disagree’, where the word ‘enjoyable’ was questioned and experiences with the system relayed using similarly positive terms of ‘interesting’, ‘useful’ and ‘informative’ (Users 19; 25; 32; 37; 49). However, around one fifth of respondents did not share this perspective (14% ‘disagreeing; 4% strongly disagreeing). For these, the lack of enjoyment originated in the disruptive effect of the BA on preconceptions around local air quality, echoing the ability of data to challenge the neighbourhood ‘halo effect’ (Boso et al. 2020; Hofflinger et al. 2019). As User 17 explained:

*I began the study feeling quite smug that living in a low populated area I thought the air quality would be consistently good – I was shocked to discover that the air quality was variable, and it made me stop and think about whether I should light the burner*.

For others, this disruption originated not in the unsettling of a preconception but in being ‘reminded’ of one they had consciously denied (User 26; also 10). Drawing attention to poor air quality—and the role of domestic burning in that—also introduced a more explicitly moral dimension to the behaviour, eliciting experiences of ‘shame’ (User 4), discomfort and ultimately ‘guilt for using the stove’ (User 36; also 43; 44; 45). Following guidance from the behaviour change literature, on the need for messages to be positive, human-centred and avoid alarmism (Riley et al. 2021), there was no design intention to elicit such responses. However, given that the system explicitly communicates the harmful effects of domestic burning, this may be unavoidable. It may also be unproblematic; only one user reporting such a response ignored the alert and, even then, cited cold weather instead of guilt as the reason for non-compliance.

The majority (29% ‘strongly agree’, 58% ‘agree’) considered the recommendations clear to understand. The traffic light system was described as ‘easy to follow’ (User 17) with ‘clear colour coding’ (User 27), while the advisory statements were reported as ‘well-presented’ (User 47), ‘informative’ (User 36), ‘short, clear and to the point’ (User 38). In this regard, the system communicated enough information without being overwhelming (Riley et al. 2021), avoiding the miscommunication found to arise through over-quantification (Heydon and Chakraborty 2022) and unclear guidance (Roberts et al. 2022).

By contrast, a smaller proportion of users (6% ‘strongly agree’; 51% ‘agree’) deemed that enough information was being communicated about the health effects of domestic combustion emissions. Those answering otherwise requested more information on impacts beyond that provided in the animation (Users 4; 35), for users to ‘refer back to later, without having to rewatch the video’ (User 19). Specifically, there was a desire for the alerts themselves to relay health effects information as text (Users 36; 37; 38; 50), supported by ‘NHS advice or peer reviewed reports’ (User 15). Given the role of this information in encouraging users to comply with alert recommendations, and the evidence of this elsewhere (D’Antoni et al. 2019), addressing the perceived shortfall of information on the health impacts of domestic burning may increase compliance going forward.

Confidence that the BA system had reduced user emissions was more dispersed compared to other measures. Just over half of participants were ‘slightly confident’ (20%) and ‘not confident at all’ (31%) that use had reduced emissions, compared with the other 48% (10% ‘completely confident’, 22% ‘fairly confident’, and 16% ‘somewhat confident’). Users explained this by recourse to their adherence to alert recommendations, where lower confidence was reported by those that did not receive red alert notifications or less consistently complied with their advice.

## Discussion

Understanding the options available to reduce PM2.5 emissions from domestic combustion is of key significance to policymakers across the UK and internationally. Triggering a polarised discussion on the possible solutions, the problem has been met with government recommendations to ‘burn better’ (Defra 2023b), which dissuades the production of smoke while encouraging emission of PM2.5, or ‘ban’ burning completely, but without considering the administrative machinery needed to support such an endeavour or exempt those experiencing fuel poverty (Mums for Lungs 2023). By foregrounding BPP’s theoretical injunction to ‘design for compliance’ (Barak-Corren and Kariv-Teitelbaum 2021: 172), this study explores a regulatory response situated between these two positions.

The findings indicate that voluntary burn alert systems have the capacity to support decision-making that discourages domestic combustion, reduces air pollution, and protects health. Widely regarded by users as clear, easy to access, interesting to use, and valued as a means of reducing emissions, the intervention scored highly on measures of usability and acceptability, speaking to the importance of user-centric design at points of entry into regulatory regimes.

Beyond validating the potential of the system, several challenges hindered consistent compliance with alert recommendations. Echoing conclusions drawn in other studies (Tarchiani et al. 2020; Haddad and de Nazelle 2018; Walker et al. 1998), a lack of confidence in the accuracy of the data undermined trust in the intervention. This had several origins, including insufficient data granularity, competing monitoring sources, and opaque sensor locations. However, as technical challenges they can be designed out. One response would be to replace the national-scale system, which relies on a sparse network of unequally distributed monitors (see Defra 2023c), with a local-level counterpart. Mirroring the geographically specific systems of the U.S.A. (see Appendix Table 4), ‘burn alerts’ could be prioritised in areas with particularly dense air quality networks, such as London (see London Air 2 2023) or Southampton (see Say 2023), where multiple official sensor networks can be drawn together to underpin alerts. This would provide more granular data and prevent the inter-network comparisons that undermined perceptions of data accuracy. Subsequently, sensor locations could be communicated as part of the postcode alert, or displayed on a map like the Sensor.Community (2023) network, to provide transparency. This would also enable local identities and norms to be activated, while allowing interventions to be supplemented by local-level awareness-raising initiatives (Tarchiani et al. 2020).

In contrast to the technical challenges, the more structural and environmental obstacles to consistent compliance cannot be designed out of the intervention. Users cited particularly cold winter temperatures and the high cost of using alternative gas or electric heating as obstructing compliance with alert recommendations not to burn. As issues external to the BA system, they speak to the context of escalating fuel costs in which this study was conducted (see Harari et al. 2023: pp 23–25). Such challenges require wider interventions, serving as a reminder that behavioural measures akin to the BA never operate in a vacuum:

*Even the most ‘perfectly’ worded messages delivered at the right time will not change a person’s behaviour if they do not have the physical or opportunity to engage. Communication needs to take place in an environment in which the social, physical and environmental barriers to behaviour change are also being addressed*.

(Riley et al. 2021: 2014)

The high fuel costs experienced by users were prompted largely by the Russian invasion of Ukraine and rising gas prices in Europe following increased post-Covid demand (Harari et al. 2023). Such obstacles may therefore be temporary. Indeed, wholesale energy prices have fallen since winter 2022 (Hinson and Bolton 2023), when users engaged with the BA system. Over the longer term, the UK government plans to phase out the installation of natural gas boilers by 2035, and completely transition away from natural gas and fossil fuel heating systems in all households by 2050, reducing future exposure to energy price fluctuations (Business, Energy and Industrial Strategy Committee 2022). Taken together, the more structural barriers to behaviour change reported here may exert less influence over alert compliance in future.

In a UK context, burn alert systems could be introduced in a voluntary capacity—as trialled here—or more formally integrated with existing domestic combustion regulations. A transition from the former to the latter could also occur, providing greater opportunity to engage the public in the introduction of a ‘just’ system of regulation (Murphy 2016). Making this shift would invoke Stage Two of BRR theory, involving more protracted testing at not only the ‘entry point’ of the interface but each subsequent level of the enforcement hierarchy (Barak-Corren and Kariv-Teitelbaum 2021:173). Taking inspiration from existing systems (see Appendix Table  4), and integrating them with the—albeit limited—SCA sanction pyramid (Defra 2022), Fig. 4 illustrates one possible form this could take.

**Fig. 4:**
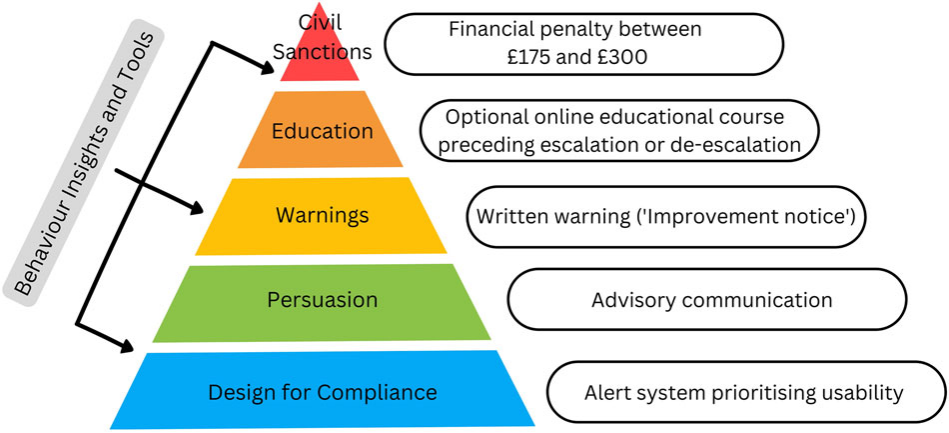
Example integration of burn alert and UK SCA enforcement hierarchy informed by BRR

Such a system would require several amendments beyond the interface itself. First, the ‘problem definition’ of ‘smoke’ would need to be modified. Currently, emission of ‘smoke’ from a chimney triggers SCA enforcement; this would have to be reinterpreted as domestic burning during a given alert. Neither Sec.20 of the Clean Air Act 1993 nor government guidance on SCA enforcement defines ‘smoke’, so this re-interpretation could be facilitated by amending either or both (Defra 2022). Second, given the UK’s divergence from WHO and EU PM2.5 limits (European Parliament 2023), an appropriate trigger threshold would need to be decided upon that avoids inconsistency between local and national governments. The action of alert triggering itself may also be automated—like the system trialled above—or manually activated by administrators. This latter approach is more common in the U.S.A but introduces a different set of design challenges (see Roberts et al. 2022), and heightens the need to establish institutional legitimacy from the perspective of the regulated (Wood et al. 2023). The third set of amendments relate to the areas in which enforcement occurs. SCA boundaries would need to be expanded to coincide with local authority jurisdictions, their monitoring density improved, and appropriate resourcing provided for enforcement (see Heydon 2023).

Following the structural impediments to behaviour change highlighted in the findings, these SCA-level changes would need to be buttressed by wider measures. Considering the role of aesthetics driving stove use outside the context of high energy prices (Emden and Murphy 2018), socio-culturally sensitive information campaigns on the public health risks of domestic combustion could be introduced alongside health warnings at the point of sale (Chakraborty et al. 2020; Bickerstaff 2004). In the context of aggregate emissions, direct or indirect installation limits in urban areas could also be considered (Carrington 2023). The hierarchy of sanctions would need to be accompanied by exemptions for those without alternatives, and support for households to move towards less expensive and environmentally impactful forms of domestic heating. As the findings suggest, introduction of restrictions without concomitant provision of accessible and affordable heating alternatives could undermine compliance, contributing to a system where enforcement falls disproportionately on those least able to comply. Following the tenets of BRR, whichever mix of interventions were introduced all would require ongoing testing and revision over time.

### Study Limitations and Recommendations

The study has several limitations. The sample excluded those where domestic combustion is their primary heat source. This was chosen for practical and ethical reasons, but further research is needed to determine whether those without a secondary heat source would respond differently to the intervention. Similarly, participants were asked to use the BA system for 2 weeks. Given that uptake of comparable interventions, such as smart metres (see Vassileva and Campillo 2016), can wane after an initial introductory period, further research is needed to determine how far voluntary engagement would continue past this point. The sample was also self-selecting, relatively small, and non-representative (see Table 3). Though it is not clear what a representative sample of domestic burners would look like (Wood et al. 2023), further research should consider using fully powered randomised control trials to evaluate effectiveness in a more diverse sample. Following Barak-Corren (2022), it would also be fruitful to explore how the intervention is experienced by different cultural and ethnic groups.

The national scope of the system ensured a greater range of users could participate, including from rural and urban settings, but was also limited in its ability to speak to aspects of local identity. The role of this in behaviour change is well-established, whereby mass communication channels are often lacking in personal and targeted messages (Mehiriz and Gosselin 2019), and thus fail to capitalise on the opportunities afforded by tailoring information to the receiver and activating local identities (Riley et al. 2021; Tarchiani et al. 2020). Despite providing air quality information on the basis of postcodes, it could not draw on these other aspects of the locality to encourage behaviour change. Further research into how national-level systems could more effectively leverage local identities is therefore recommended, alongside research into how local-level systems can effectively operationalise this aspect of their communications.

The BA system provided real-time air quality readings and was not predictive. Despite recommendations that lead-time be provided for users (Roberts et al. 2022), to enable space for decisions to be made about future behaviour, this was beyond the scope of the project. The lack of a smartphone app also meant that ‘push notifications’ could not be included, as similarly recommended (Potter et al. 2021; Kaltenberger et al. 2020). Notwithstanding the resources needed to introduce these features, exploring their effect on compliance is another avenue for further exploration.

Finally, compliance with existing burn alert systems may be more reliant on wider educational interventions than is first apparent. The Federal system of government in the U.S.A. devolves more power and resources to the local level compared to the unitary model of the UK. As a result, monitoring networks are denser, public engagement initiatives more established and wide-ranging, and burn alert systems more embedded in regimes targeting pollution from sources beyond domestic combustion (see Bay Area Air Quality Management District 2024). It would therefore be helpful for future research to explore the effect of these less coercive instruments on compliance with burn alerts narrowly, but also decisions around domestic combustion more generally.

## Conclusion

With domestic combustion posing an air quality problem for the UK and beyond, the importance of successful interventions that encourage people to reduce these emissions is imperative. This article has drawn on BRR to combine the air pollution communication and extreme weather warning literatures to ‘design for compliance’—and trial—the UK’s first burn alert system with this purpose. Highlighting the potential for voluntary ‘burn alerts’ to change behaviour, the study has also drawn attention to the importance of context when considering the limits to design efforts aimed at encouraging consistent compliance. In doing so, the article has demonstrated how BRR’s theoretical prescription to ‘design for compliance’ can be practically operationalised, and evidenced how existing regulations could be modified to intervene more directly in PM2.5 emissions from domestic combustion in the future.

## Data Availability

The data that supports the findings of this study are available from the authors upon reasonable request.

## Appendix

Tables 4–6

**Table 4:** U.S.A. burn alert system characteristics

Location and system name	Alert trigger	Escalating compliance measures	Exemptions	Accompanying measures
Bay Area Air Quality Management District ‘Spare the Air Alerts’	Levels forecast to breach 35 ug/m^3^ PM2.5 on 24-h average	(1) Notice of Violation (+ opportunity to comply); (2) Penalty (1st time non-compliance $100); (3) Penalty (repeat non-compliance $500); (4) Increasing penalties; (5) Criminal sanction	Only heat source; Non-functioning primary heat source	Blanket prohibition on ‘excessive smoke’ (No. 1 Ringlemann Chart) Rebate programme for upgrading stoves; Installation ban in new homes; Ban on sale, advertising, install or swap of non-EPA approved stoves;
Puget Sound Clean Air Agency ‘Burn Bans’	Stage 1) Levels forecast to breach 30 ug/m^3^ on 24 h average within 72 h; Stage 2) Triggered if Stage 1 has not reduced PM2.5; PM2.5 levels exceed 30 ug/m^3^ on 24 h average; May be called without Stage 1 if, *et alia*, PM2.5 forecast to exceed 35 ug/m^3^	(1) Notice of Violation (2) Penalty (1st time non-compliance, $200 fine conditionally waived if agreement signed not to repeat, take ‘clean burn test’ and sign up for ‘burn ban’ notifications); (3) Penalty (2nd time non-compliance. No options to reduce fine. $200 original + $500 new fine. Repeat if continued non-compliance)	Stage 1) EPA-Certified stoves; Only heat source + permitted exemption; Stage 2) Only heat source	Blanket prohibition on ‘excessive smoke’ (No. 1 Ringlemann Chart) During burn bans all outdoor burning prohibited; Stove scrappage scheme ($350 for removal and recycling of appliance)
South Coast Air Quality Management District ‘Check Before You Burn’	November through February, mandatory no-burn alerts issued when PM2.5 forecast to exceed 29 ug/m^3^	(1) Notice of Violation (2) Wood smoke awareness course (1st time non-compliance or $50 penalty) (3) Penalty (2nd time non-compliance $150 fine) (4) Penalty (3rd time non-compliance, $500 fine or implementation of environmentally beneficial project as derived through the mutual settlement process	Only heat source; Homes above 3,000 feet elevation and certain valleys; Low income homes	Blanket prohibition on installing appliances in new developments; Can only be installed in existing developments as secondary heat source

**Table 5:** Avoidance response by alert type crosstabulation (*n* = 50)

Avoided lighting because of an Amber alert								
		I never received an Amber burner alert	Always	Often	Sometimes	Rarely	Never	Total
Avoided lighting because of a red alert	I never received a red burner alert	8.0%	10.0%		4.0%	8.0%	14.0%	44.0%
	Always		4.0%		2.0%	4.0%		10.0%
	Often	2.0%		4.0%				6.0%
	Sometimes	2.0%			4.0%	6.0%	2.0%	14.0%
	Rarely	4.0%					4.0%	8.0%
	Never	8.0%				4.0%	6.0%	18.0%
Total		24.0%	14.0%	4.0%	10.0%	22.0%	26.0%	100.0%

**Table 6:** Response frequencies by alert type

	Frequency	Percent	Valid percent	Cumulative percent
Avoided lighting because of a red alert				
I never received a red alert	22	44.0	44.0	44.0
Always	5	10.0	10.0	54.0
Often	3	6.0	6.0	60.0
Sometimes	7	14.0	14.0	74.0
Rarely	4	8.0	8.0	82.0
Never	9	18.0	18.0	100.0
Total	50	100.0	100.0	
Avoided lighting because of an Amber alert				
I never received an Amber alert	12	24.0	24.0	24.0
Always	7	14.0	14.0	38.0
Often	2	4.0	4.0	42.0
Sometimes	5	10.0	10.0	52.0
Rarely	11	22.0	22.0	74.0
Never	13	26.0	26.0	100.0
Total	50	100.0	100.0	
